# In vivo dissection of the mouse tyrosine catabolic pathway with CRISPR-Cas9 identifies modifier genes affecting hereditary tyrosinemia type 1

**DOI:** 10.1093/genetics/iyae139

**Published:** 2024-08-23

**Authors:** Jean-François Rivest, Sophie Carter, Claudia Goupil, Pénélope Antérieux, Denis Cyr, Roth-Visal Ung, Dorothée Dal Soglio, Fabrice Mac-Way, Paula J Waters, Massimiliano Paganelli, Yannick Doyon

**Affiliations:** Centre Hospitalier Universitaire de Québec Research Center and Faculty of Medicine, Laval University, Québec City, QC G1V 4G2, Canada; Université Laval Cancer Research Centre, Québec City, QC G1V 0A6, Canada; Centre Hospitalier Universitaire de Québec Research Center and Faculty of Medicine, Laval University, Québec City, QC G1V 4G2, Canada; Université Laval Cancer Research Centre, Québec City, QC G1V 0A6, Canada; Centre Hospitalier Universitaire de Québec Research Center and Faculty of Medicine, Laval University, Québec City, QC G1V 4G2, Canada; Université Laval Cancer Research Centre, Québec City, QC G1V 0A6, Canada; Centre Hospitalier Universitaire de Québec Research Center and Faculty of Medicine, Laval University, Québec City, QC G1V 4G2, Canada; Université Laval Cancer Research Centre, Québec City, QC G1V 0A6, Canada; Medical Genetics Service, Dept. Laboratory Medicine and Dept. Pediatrics, Centre Hospitalier Universitaire de Sherbrooke (CHUS), Sherbrooke, QC J1H 5N4, Canada; Centre Hospitalier Universitaire de Québec Research Center and Faculty of Medicine, Laval University, Québec City, QC G1V 4G2, Canada; Centre Hospitalier Universitaire Sainte-Justine Research Center, Université de Montréal, Montréal, QC H3T 1C5, Canada; Centre Hospitalier Universitaire de Québec Research Center and Faculty of Medicine, Laval University, Québec City, QC G1V 4G2, Canada; Medical Genetics Service, Dept. Laboratory Medicine and Dept. Pediatrics, Centre Hospitalier Universitaire de Sherbrooke (CHUS), Sherbrooke, QC J1H 5N4, Canada; Centre Hospitalier Universitaire Sainte-Justine Research Center, Université de Montréal, Montréal, QC H3T 1C5, Canada; Centre Hospitalier Universitaire de Québec Research Center and Faculty of Medicine, Laval University, Québec City, QC G1V 4G2, Canada; Université Laval Cancer Research Centre, Québec City, QC G1V 0A6, Canada

**Keywords:** Hereditary tyrosinemia type 1, CRISPR-Cas9, in vivo genome editing, mouse, tyrosine catabolic pathway, Genetic Models of Rare Diseases

## Abstract

Hereditary tyrosinemia type 1 is an autosomal recessive disorder caused by mutations (pathogenic variants) in fumarylacetoacetate hydrolase, an enzyme involved in tyrosine degradation. Its loss results in the accumulation of toxic metabolites that mainly affect the liver and kidneys and can lead to severe liver disease and liver cancer. Tyrosinemia type 1 has a global prevalence of approximately 1 in 100,000 births but can reach up to 1 in 1,500 births in some regions of Québec, Canada. Mutating functionally related “modifier’ genes (*i.e*. genes that, when mutated, affect the phenotypic impacts of mutations in other genes) is an emerging strategy for treating human genetic diseases. In vivo somatic genome editing in animal models of these diseases is a powerful means to identify modifier genes and fuel treatment development. In this study, we demonstrate that mutating additional enzymes in the tyrosine catabolic pathway through liver-specific genome editing can relieve or worsen the phenotypic severity of a murine model of tyrosinemia type 1. Neonatal gene delivery using recombinant adeno-associated viral vectors expressing *Staphylococcus aureus* Cas9 under the control of a liver-specific promoter led to efficient gene disruption and metabolic rewiring of the pathway, with systemic effects that were distinct from the phenotypes observed in whole-body knockout models. Our work illustrates the value of using in vivo genome editing in model organisms to study the direct effects of combining pathological mutations with modifier gene mutations in isogenic settings.

## Introduction

Inborn errors of metabolism (IEMs) are inherited genetic disorders caused by disruptions in a specific enzymatic reaction within a metabolic pathway that induce pathology through toxic metabolite accumulation or deficiencies in downstream metabolites (Lanpher *et al*. 2006). First described by Archibald Garrod over 100 years ago, IEMs are frequently considered to be monogenic diseases (Scriver 2008). However, this classification may over-simplify the biological reality, as patients often present a spectrum of phenotypes (Scriver and Waters 1999; Dipple and McCabe 2000; Argmann *et al*. 2016). Importantly, modifier genes can profoundly influence the phenotype associated with mutation(s) (pathogenic variants) at a primary “disease-causing” gene locus (Scriver and Waters 1999; Dipple and McCabe 2000; Génin *et al*. 2008; Harper *et al*. 2015; Argmann *et al*. 2016). Genome editing therapies targeting modifier genes are already showing great promise in clinical trials, particularly for hemoglobinopathies (Bauer *et al*. 2013; Canver *et al*. 2015; Frangoul *et al*. 2021). Liver-specific base editing of *PCSK9* is also currently being investigated in a clinical trial to treat familial hypercholesterolemia (Rothgangl *et al*. 2021; Lee *et al*. 2023). Thus, identifying and characterizing modifier genes can provide valuable mechanistic information and spark the development of novel therapeutics.

**Fig. 1.**
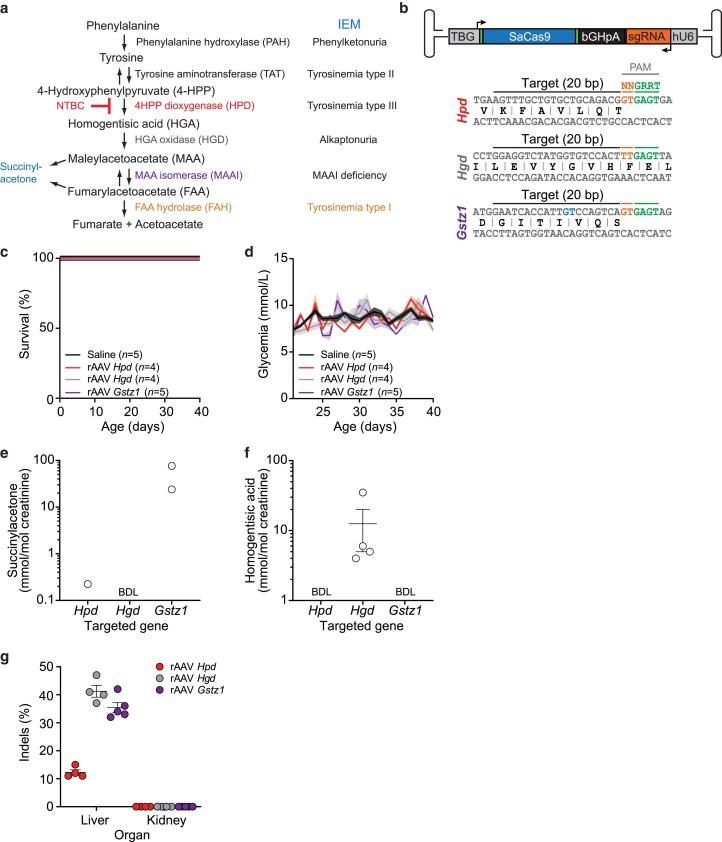
In vivo genome editing of the tyrosine catabolic pathway by rAAV8-SaCas9 in C57BL/6N mice. a) The tyrosine degradation pathway and associated inborn errors of metabolism (IEMs). The catabolic reaction inhibited by NTBC is indicated in red. 4-HPP, 4-hydroxyphenylpyruvate; HGA, homogentisic acid; MAA, maleylacetoacetate; FAA, fumarylacetoacetate. b) Top: Schematic of the rAAV-SaCas9 vector. The thyroxine-binding globulin (TBG), bovine growth hormone polyadenylation (bGHpA), and hU6 promoter sequences are indicated. Bottom: Sequences of the sgRNAs targeting *Hgd, Hgd,* and *Gstz1* chosen for in vivo studies. The protospacer adjacent motifs (PAMs) are annotated. The two blue nucleotides in the *Gstz1* target site are mismatches compared to the human sequence. c) Neonatal male C57BL/6N pups were injected with 5 × 10^10^ vector genomes (VGs) of rAAV8-SaCas9 into the retro-orbital sinus, weaned at 21 days old, and sacrificed at 40 days of age. Mice were assayed for phenotypic and metabolic modifications after weaning. Kaplan-Meier survival curves of male C57BL/6N mice injected at 2 days old with 5 × 10^10^ VGs rAAV8-SaCas9 or saline into the retro-orbital sinus. The numbers of mice per group (*n*) and rAAV8 targets are indicated. d) Glycemia of non-fasted mice. Solid lines indicate the mean and the shaded areas denote the SEM. e) Urine succinylacetone and f) homogentisic acid levels in mice treated as in c) were determined 35 days after weaning. Samples were collected over 24 hours using metabolic cages containing 2–3 mice (Supplementary Tables 2 and 3). Also indicated for each group of animals are the mean and the standard error of the mean (SEM). The detection limits for succinylacetone and homogentisic acid were 0.1 mmol/mol and approximately 3 mmol/mol creatinine, respectively. BDL: below detection limit. g) Genomic whole-liver and kidney DNA was extracted, and Surveyor assays were used to determine indel frequencies. Each symbol represents a different animal. Also indicated for each group of animals are the mean and the standard error of the mean (SEM). A mouse injected with saline was used as the negative control for the Surveyor assay.

The phenylalanine and tyrosine degradation pathway is notable historically as it is linked to the description of the first inborn error of metabolism, alkaptonuria (Garrod 1902). Loss of function of each enzyme in this pathway leads to a different IEM (Fig. 1a). In the first step, phenylalanine is converted to tyrosine by phenylalanine hydroxylase (PAH). Loss of PAH activity leads to phenylketonuria; a disease characterized by intellectual disability and seizures (OMIM 261600). Tyrosine is then converted to 4-hydroxyphenylpyruvate (4-HPP) by tyrosine aminotransferase (TAT). TAT inactivation results in tyrosinemia type II, which causes painful corneal lesions, skin disease, and intellectual disability (OMIM 276600). In the next step, 4-HPP is converted to homogentisic acid (HGA) by 4-hydroxyphenylpyruvate dioxygenase (HPD). Its loss of function results in tyrosinemia type III, a disease characterized by intellectual disability, seizures, and intermittent ataxia (OMIM 276710). In the fourth reaction, HGA is converted to maleylacetoacetate (MAA) by homogentisate 1,2-dioxygenase (HGD). Patients with inactive HGD display high HGA levels and develop alkaptonuria, the prototypical IEM described by Garrod (Scriver 2008), which results in arthritis, heart valve and kidney disease, and pigmentation changes to the cartilage and urine (OMIM 203500). In the penultimate step in tyrosine degradation, MAA is converted to fumarylacetoacetate (FAA) by glutathione S-transferase zeta 1 (GSTZ1), also known as maleylacetoacetate isomerase (MAAI). Individuals with GSTZ1 deficiency display mild hypersuccinylacetonemia which appears to be clinically insignificant [Yang *et al*. 2017; van Vliet *et al*. 2023 (OMIM 617596)]. Finally, fumarylacetoacetate hydrolase (FAH) converts FAA into fumarate and acetoacetate, which are used for energy production by the tricarboxylic acid cycle and reconverted to acetyl-CoA, respectively.

Among IEMs affecting this pathway, one of the most severe is undoubtedly hereditary tyrosinemia type 1 (HT-I), a disorder caused by the absence of a functional *FAH* allele [OMIM 276700 (Lindblad *et al*. 1977)]. Loss of FAH activity can lead to hepatic failure with renal and neurological comorbidities, along with a high risk of developing hepatocellular carcinoma (Chinsky *et al*. 2017; Tanguay *et al*. 2017). HT-I results from the accumulation of tyrosine and its toxic metabolites, such as FAA, MAA, and succinylacetone (SA), a byproduct of both FAA and MAA degradation and the diagnostic marker for HT-I (Morrow and Tanguay 2017). However, the molecular mechanisms by which these compounds damage the liver and kidneys are poorly characterized (Chinsky *et al*. 2017; Morrow and Tanguay 2017). HT-I is progressive and life-threatening if left untreated. Standard care includes diet therapy to limit phenylalanine and tyrosine intake and lifelong treatment with 2-(2-nitro-4-trifluoromethylbenzoyl)-1,3-cyclohexanedione (NTBC; also known as nitisinone), a potent inhibitor of the upstream enzyme HPD (Alvarez *et al*. 2017), to prevent toxic metabolite accumulation in the liver and kidneys [Grompe *et al*. 1995; Lindstedt *et al*. 1992; Larochelle *et al*. 2012 (Fig. 1a)]. Importantly, non-compliance with NTBC and diet treatment is a serious challenge for the clinical management of HT-I and results in higher risks of patients developing hepatocellular carcinoma, as well as painful corneal lesions due to high circulating tyrosine levels and neurological crises due to high SA levels (Aktuglu-Zeybek *et al*. 2017). While this catabolic pathway is well characterized at the molecular genetic level, the broad spectrum of phenotypes and disease severity observed in patients with HT-I—even within families—is not completely understood (Mitchell and Yang 2017).

Several knockout mouse models have been created to evaluate the interplay between the different genes in the phenylalanine and tyrosine degradation pathway and their impacts on various disease phenotypes (Grompe *et al*. 1993; Shedlovsky *et al*. 1993; Montagutelli *et al*. 1994; Endo *et al*. 1995; Fernández-Cañón *et al*. 2002). The *Fah*^Δexon5^ (*Fah*^−/−^) mouse is a well-established murine model for HT-I pathophysiology (Grompe *et al*. 1993, 1995). In these mice, neonatal lethality can be prevented by early treatment with NTBC, which completely corrects the metabolic liver disease and results in a phenotype analogous to human tyrosinemia type III (Grompe *et al*. 1995). Upon NTBC withdrawal, however, disease progression resumes leading to death in a short period of time (Grompe 2017). This experimental system offers an opportunity to test treatment options in vivo. Moreover, in both mice and humans, transplanted or genetically corrected hepatocytes have a potent selective growth advantage in the mutant liver lowering the initial threshold for treatment efficacy (Chen *et al*. 2000; Grompe 2017, 1993; Held *et al*. 2005; Montini *et al*. 2002; Overturf *et al*. 1996; Paulk *et al*. 2012a, 2010). Hence, this model has become a staple for hepatic gene therapy research (Paulk *et al*. 2012a, 2010; Grompe 2017). Whole*-*body double knockout mouse studies have revealed that full metabolic blocks at different points upstream in the pathway greatly affect the disease phenotype. For example, while *Fah*^−/−^  *Hpd*^−/−^ and *Fah*^−/−^  *Hgd*^−/−^ animals survived without NTBC, *Fah^−/−^ Gstz1^−/−^* mice displayed an even more severe phenotype than *Fah^−/−^* mice (Endo *et al*. 1997; Manning *et al*. 1999; Fernández-Cañón *et al*. 2002) (Fig. 1a). Accordingly, both short hairpin RNA (shRNA)-mediated knockdown and CRISPR-Cas9-directed inactivation of *Hpd* in the liver of *Fah^−/−^* animals enabled the survival after NTBC withdrawal, suggesting that metabolically blocking the pathway in the liver is sufficient for a systemic therapeutic effect (Ibraheim *et al*. 2018; Pankowicz *et al*. 2016; Agudelo *et al*. 2020; Gu *et al*. 2021). Still, the impact of liver-specific inactivation of *Hgd* and *Gstz1* via in vivo genome editing in the HT-I mouse model has yet to be described. To test this, we used a hepatocyte-specific promoter and rAAV8-mediated delivery of *Streptococcus aureus* Cas9 (SaCas9) (Gao *et al*. 2002; Nakai *et al*. 2005; Carrillo-Carrasco *et al*. 2010; Ran *et al*. 2015) to inactivate *Hpd*, *Hgd*, and *Gstz1* in the liver of neonatal *Fah*^Δexon5^ (*Fah*^−/−^) mice (Fig. 1a). We did not target *Tat*, as its loss produces a more severe phenotype in mice than the loss of *Hpd*, *Hgd*, and *Gstz1*, according to phenotyping data from the International Mouse Phenotyping Consortium (Grompe *et al*. 1993; Endo *et al*. 1995; Manning *et al*. 1999; Fernández-Cañón *et al*. 2002; Groza *et al*. 2023). We found that, while targeting *Hpd* and *Gstz1* yielded outcomes like those observed in whole-body double mutant mice, targeting *Hgd* led to systemic and lethal effects in contrast to the genetic suppression observed in the conventional *Fah^−/−^ Hgd^−/−^* model. This work highlights the value of somatic genome editing in animals for modeling human disorders (Alves-Bezerra *et al*. 2019).

## Materials and methods

### Genome editing vectors

The Cytomegalovirus-driven SaCas9 nuclease vector pX601 (Ran *et al*. 2015) (Addgene plasmid #61591) was a gift from Feng Zhang (Massachusetts Institute of Technology). Target sequences for *Hpd*, *Hgd*, and *Gstz1* were designed using the web-based CRISPR design tool CRISPOR (Haeussler *et al*. 2016). The sgRNA sequences used are listed in Supplementary Table 1. When required, the sgRNA sequence was modified to encode a G at position 1, to meet the transcription initiation requirement of the human U6 promoter. Following in vitro screening, selected SaCas9 sgRNAs were cloned into the thyroxine-binding globulin (TBG)-driven SaCas9 nuclease rAAV vector pX602 (Ran *et al*. 2015) (Addgene plasmid #61593; also a gift from Feng Zhang) for in vivo gene editing. The inverted terminal repeat integrity of the rAAV vector pX602 was assessed by BssHII digestion.

### Cell culture and transfection

Neuro2A cells were obtained from the ATCC (CCL-131, Manassas, VA, USA) and maintained at 37°C under 5% CO_2_ in Dulbecco's modified Eagle's medium (DMEM, high glucose, GlutaMAX™ Supplement) (#10566016, Thermo Fisher Scientific, Waltham, MA, USA) supplemented with 10% fetal bovine serum (FBS; #12483020, Gibco, Thermo Fisher Scientific, Waltham, MA, USA), 1% penicillin-streptomycin (#15140122, Thermo Fisher Scientific, Waltham, MA, USA). The cells were tested and found negative for mycoplasma contamination. Cells (2 × 10^5^) were transfected with 500 ng of pX601 (expressing both the sgRNA and SaCas9) using an Amaxa 4D-Nucleofector (Lonza, Basel, Switzerland) according to the manufacturer's recommendations and harvested 3 days post-transfection.

### Surveyor and tracking of indels by decomposition assays

Genomic DNA was extracted from 2.5 × 10^5^ Neuro2A cells with 250 µL of QuickExtract DNA Extraction Solution (#QE09050, Lucigen, Middleton, WI, USA) or from 30 mg of mouse liver using an EZ-10 Spin Column Animal Genomic DNA Miniprep Kit (#BS628, Bio Basic, Markham, ON, CA), per the manufacturers’ recommendations. Loci were amplified by polymerase chain reaction (PCR) using the primers listed in Supplementary Table 7. Surveyor assays were performed with the Surveyor Mutation Detection Kit (#706020, Integrated DNA Technologies, Coralville, IA, USA) as described (Guschin *et al*. 2010). Samples were resolved on 10% polyacrylamide gels in Tris–borate–EDTA buffer and bands were visualized with RedSafe Nucleic Acid Staining Solution (#21141, iNtRON Biotechnology, Seongnam, South Korea). Gels were imaged using a ChemiDoc MP Imaging System (Bio-Rad, Hercules, CA, USA) and bands were quantified using Image Lab Software (Bio-Rad). Tracking of indels by decomposition (TIDE) analysis was performed using a significance threshold value for decomposition of *P* < 0.001 (Brinkman *et al*. 2014).

### Adeno-associated virus production

The rAAV8 s were produced by the Canadian Neurophotonics Platform's Viral Vector Core (The Molecular Tools Platform) using the triple plasmid transfection method, as described (Gray *et al*. 2011). Briefly, HEK293T17 (ATCC CRL-11268, Manassas, VA, USA) cells were transfected using polyethylenimine (#23966, Polysciences, Warrington, PA, USA) with the helper plasmid pxx-680 (a gift from R. Jude Samulski, University of North Carolina), the rep/cap hybrid plasmid pAAV2/8 (Addgene #112864, a gift from James Wilson, University of Pennsylvania), and the rAAV vector pX602. After 24 hours, the medium was replaced with medium without FBS, and the cells were harvested 24 hours later. rAAV particles were purified from the cell extracts using freeze/thaw lysis followed by a discontinuous iodixanol gradient. Viruses were resuspended in phosphate-buffered saline containing 320 mM NaCl, 5% D-sorbitol, and 0.001% pluronic acid (F-68), aliquoted, and stored at −80°C. The rAAVs were titrated by quantitative PCR using LightCycler 480 SYBR Green I Master mix (#04707516001, Roche, Basel, Switzerland) and inverted terminal repeat primers as described (Aurnhammer *et al*. 2012). Vector yields were 1 × 10^13^–3 × 10^13^ VG/mL. The purity of viral preparations was determined by sodium dodecyl sulfate (SDS)-–polyacrylamide gel electrophoresis on a 4–15% Mini-PROTEAN TGX Stain-Free Gel (#4568084, Bio-Rad, Hercules, CA, USA) in Tris–glycine–SDS buffer (Supplementary Fig. 3).

### Animal experiments

*Fah^−/−^* mice (Grompe *et al*. 1993) with a C57BL/6N genetic background were a kind gift from Robert Tanguay (Laval University). C57BL/6N mice were purchased from Charles River (Strain code 027) (Wilmington, MA, USA). All mice were group-housed and fed a standard chow diet (Harlan #2018SX) with free access to food and water. For *Fah^−/−^* mice, the drinking water was supplemented with 7.5 mg/L NTBC. Mice were exposed to a 12:12-h dark-light cycle and kept at an ambient temperature of 23 ± 1°C. Animals were cared for and handled according to the *Canadian Guide for the Care and Use of Laboratory Animals.* The Laval University Animal Care and Use Committee approved the procedures.

Neonatal (2-day-old) pups were injected intravenously in the retro-orbital sinus (Yardeni *et al*. 2011) with saline or 5 × 10^10^ vector genomes (VGs) of an rAAV8, adjusted to an injection volume of 20 µL with saline. *Fah^−/−^* mice were weaned at 21 days old and NTBC was removed at the indicated time points. Body weight and glycemia were monitored post-NTBC removal. Glycemia was measured in unfasted mice between 9 and 10 AM. Animals were sacrificed by cardiac puncture under anesthesia at predetermined time points or when they had lost 20% of their body weight. Most of the liver and one kidney were snap-frozen, while a portion of the liver and the other kidney were fixed in 4% paraformaldehyde.

### Urine collection and SDS–PAGE analysis

Urine was collected from groups of 2–5 mice overnight using metabolic cages (Tecniplast) before and at different time points after NTBC removal. Urine was centrifuged at 900*×g* for 5 minutes, aliquoted, and frozen at −80°C for downstream applications. For SDS–PAGE analysis, 1 µL of urine per mouse was loaded on a 4–15% Mini-PROTEAN TGX Stain-Free Gel (#4568084, Bio-Rad, Hercules, CA, USA) before Coomassie staining with QC Colloidal Coomassie (#1610803, Bio-Rad) and imaging using a ChemiDoc MP Imaging System (Bio-Rad).

### Histology

Liver portions and kidneys were fixed in 4% paraformaldehyde for 24 hours, then dehydrated in 70% ethanol, embedded in paraffin, and sliced into 4-µm sections, which were mounted on slides. Liver sections were stained with hematoxylin and eosin and reviewed by a blinded pathologist and a blinded hepatologist at a magnification of 200X. Kidney sections were stained with Masson's trichrome and reviewed by a blinded nephrologist using an Olympus BX45 microscope at different magnifications.

### SA and HGA quantification

Urine SA and HGA levels were quantified by gas chromatography-mass spectrometry (GC-MS), as previously described (Sweetman 1991; Cyr *et al*. 2006). All analyses were performed in the biochemical genetics laboratory at the CHUS.

### Statistical analysis

**Fig. 2.**
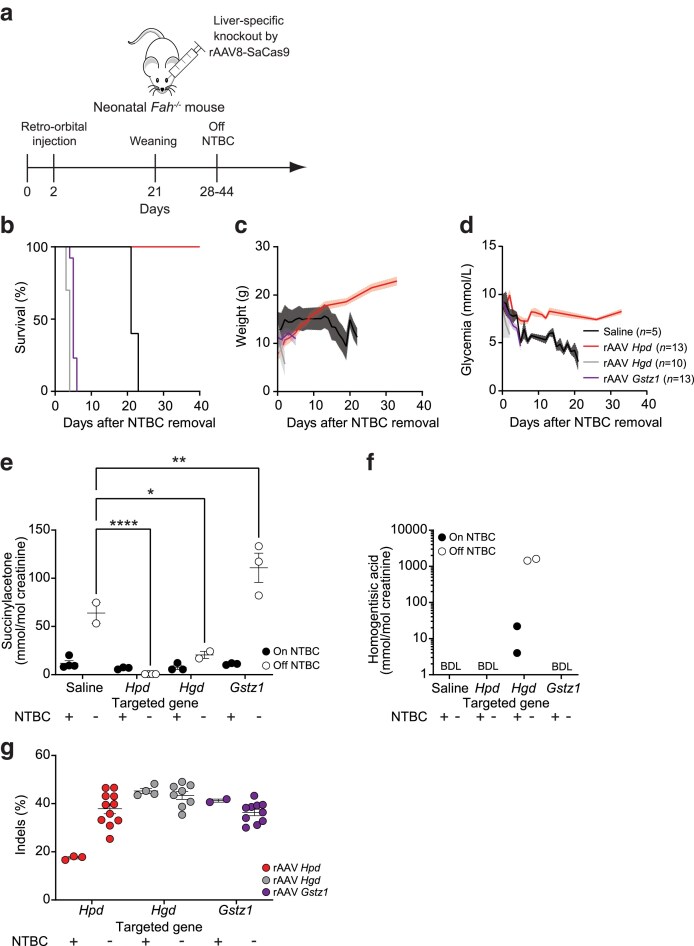
Metabolic rewiring through liver-specific knockout of *Hpd, Hgd,* and *Gstz1* in *Fah^−/−^* mice. a) Experimental design for in vivo editing. Neonatal (2-day-old) male *Fah^−/−^* pups were injected with 5 × 10^10^ VGs of rAAV8-SaCas9 or saline into the retro-orbital sinus and weaned at 21 days old. NTBC treatment was stopped at different time points; 28 days (saline, *n* = 3; *Hpd, n* = 7; *Hgd*, *n* = 3; *Gstz1*, *n* = 3), 30 days (*Hpd, n* = 6; *Gstz1*, *n* = 3), 41 days (saline, *n* = 2; *Gstz1*, *n* = 7), or 44 days (*Hgd*, *n* = 7). Since the period elapsed between weaning and NTBC removal did not affect the outcome, mice from each treatment group were combined in the graphs shown in (b-d). The numbers of mice per group (*n*) and rAAV targets are indicated. b) Kaplan-Meier survival curves following NTBC removal. Mice were sacrificed after losing 20% of their body weight. All animals injected with rAAV8-SaCas9 experienced statistically significant differences in survival as determined per the log-rank test when compared to a group of saline-injected animals, whose median survival was 21 days: *Hpd*-treated animals, complete survival (*P* < 0.0001); *Hgd*-treated animals, median survival of 4 days (*P* < 0.001), *Gstz1*-treated animals, median survival of 5 days (*P* < 0.001). c) Body weight was measured daily following NTBC removal. Solid lines indicate the mean and shaded areas denote the standard error of the mean (SEM). d) Glycemia was monitored in non-fasted mice. e) Urine succinylacetone and f) homogentisic acid levels were determined 24 hours before NTBC removal and again 24 hours before the predicted time of sacrifice (1, 4, and 19 days post-NTBC removal for mice treated with *Hgd* rAAV8, *Gstz1* rAAV8, and saline, respectively. Levels were determined in *Hpd* rAAV8-treated mice 34 days after NTBC removal. Samples were collected over 24 hours using metabolic cages containing 2–5 mice (Supplementary Tables 4 and 5). Also indicated for each group of animals are the mean and the standard error of the mean (SEM). Statistical analysis was performed with 2-way ANOVA followed by Tukey tests for the represented pairwise comparisons. * : *P* < 0.05; ** : *P* < 0.01; **** : *P* < 0.0001. The detection limits for succinylacetone and homogentisic acid were 0.1 mmol/mol and approximately 3 mmol/mol creatinine, respectively. BDL: below detection limit. g) Genomic DNA was extracted from whole livers of mice treated with *Hpd*-, *Hgd-* and *Gstz1-*targeting vectors sacrificed either at 30 days of age with continuous NTBC treatment or at time of sacrifice after NTBC removal (respectively one year for *Hpd*-targeted animals, 3–4 days for *Hgd*-targeted animals and 4–6 days for *Gstz1*-targeted animals), and Surveyor assays were used to determine indel frequencies. Each symbol represents a different animal. Also indicated for each group of animals are the mean and the standard error of the mean (SEM). A mouse injected with saline was used as the negative control for the Surveyor assay.

**Fig. 3.**
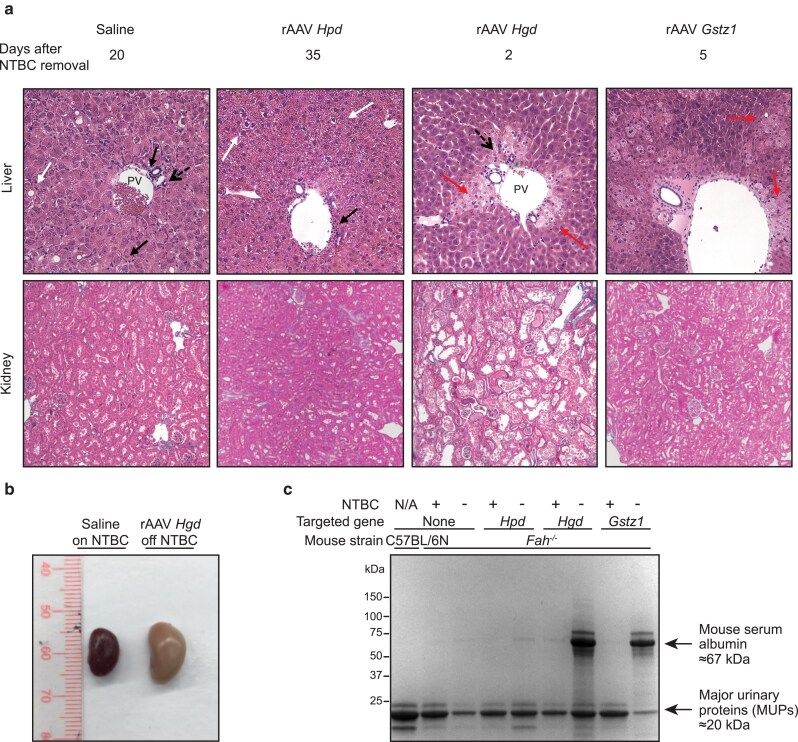
Tissue analysis of Fah−/− mice following metabolic rewiring by rAAV8-SaCas9. a) Kidney and liver sections from Fah−/− mice treated with saline or rAAV8-SaCas9 vectors targeting Hpd, Hgd, or*Gstz1* as in Fig. 2. *Fah^−/−^* mice injected with saline and the *Hpd, Hgd,* and *Gstz1* rAAV8 s were sacrificed 20, 35, 2, and 5 days after NTBC removal, respectively. Top panel: Representative Masson's trichrome-stained kidney sections from the treatment groups. Magnification: 10×. Bottom panel: Representative hematoxylin and eosin-stained liver sections from the treatment groups. PV: portal vein; black arrow: portal and lobular inflammation; dashed arrow: ductular proliferation; white arrow: necrosis; red arrow: ballooning degeneration. Magnification: 200×. b) Representative kidneys from *Fah^−/−^* mice that were treated with saline and kept on NTBC (left) or treated with a vector targeting *Hgd* with NTBC withdrawn (right). c) Urine samples (1 µl per well) from treated *Fah^−/−^* animals on (+) and off (−) NTBC and untreated controls (as described in Fig. 2) were loaded on a 4–15% gradient mini-PROTEAN TGX Stain-Free gel before electrophoresis, Coomassie staining, and imaging. Urine from a C57BL/6N male mouse was used as a negative control. A band of the size expected for mouse serum albumin is indicated with an arrow. Also indicated with an arrow are bands of the expected size for the mouse major urinary proteins (MUPs).

Statistical tests were performed when appropriate and graphs were drawn using Prism (GraphPad) version 10 software. Mean and standard error of the mean (SEM) are shown where indicated. Kaplan-Meier survival curves were used to display the survival of the *Fah^−/−^* and C57BL/6N animals following NTBC removal (Figs. 2b and 3, a and and 4a), and the log-rank test was used when appropriate to compare each treated group to a group of saline-injected animals. For analysis of urine succinylacetone values (Fig. 2e), normal distribution was assessed using the Shapiro-Wilk test before analysis of variance (two-way ANOVA) and Tukey tests for the represented pairwise comparisons. * : *P* < 0.05; ** : *P* < 0.01; **** : *P* < 0.0001.

**Fig. 4.**
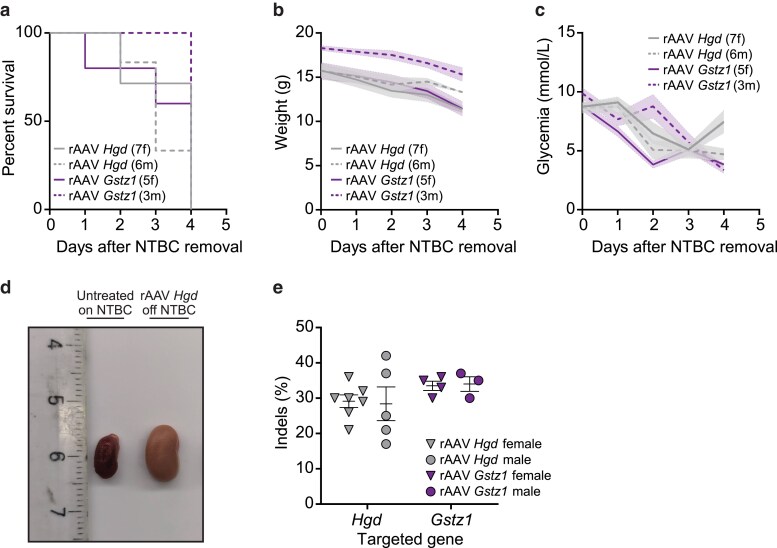
Metabolic rewiring by inactivation of Hgd or Gstz1 yields similar effects in both female and male Fah−/− mice. a) Neonatal pups of both sexes were injected at birth with 5 × 1010 VGs of rAAV8-SaCas9 and NTBC was withdrawn at 28 days of age. Mice were sacrificed after losing 20% of their body weight. Kaplan-Meier survival curves of female (f; solid lines) and male (m; dotted lines) Fah−/− animals following NTBC withdrawal. b) Body weight was measured daily following NTBC removal. Solid or dotted lines indicate the mean and shaded areas denote the standard error of the mean (SEM). c) Glycemia was monitored in non-fasted mice. d) Representative kidneys from female Fah−/− mice of a similar age that were either untreated and kept on NTBC (left) or treated with a vector targeting Hgd (right) after NTBC withdrawal. e) Genomic DNA was extracted from whole livers of mice treated with Hgd- and Gstz1-targeting vectors meeting the sacrifice endpoint, and Surveyor assays were used to determine indel frequencies. Each symbol represents a different animal. Female animals are represented as triangles, whereas males are represented as circles. Also indicated for each group of animals are the mean and the standard error of the mean (SEM). A mouse injected with saline was used as the negative control for the Surveyor assay.

## Results

### Potent in vivo editing in the liver of neonatal C57BL/6n mice rewires the tyrosine catabolic pathway

Since most of the enzymes in the tyrosine catabolic pathway are expressed mainly in the liver and kidneys, we hypothesized that liver-specific gene disruption would produce a systemic impact on metabolic functions. This can be achieved in neonatal mice using CRISPR-Cas9 delivered via rAAV8 injected systemically, which preferentially, but not exclusively, transduce mouse hepatocytes (Gao *et al*. 2002; Nakai *et al*. 2005). We further constrained the expression of SaCas9 to hepatocytes by using the liver-specific TBG promoter (Carrillo-Carrasco *et al*. 2010; Ran *et al*. 2015) (Fig. 1b). The single guide RNA (sgRNA) targeting a specific gene is driven by the ubiquitous U6 promoter (Ran *et al*. 2015). Following target cleavage in mouse hepatocytes, DNA repair mainly occurs through the error-prone non-homologous end-joining pathway (NHEJ), creating insertions and deletions (indels) that disrupts the target gene (Porteus 2019).

Using CRISPOR (Haeussler *et al*. 2016), we designed several SaCas9 sgRNAs against *Hpd, Hgd,* and *Gstz1* (the gene encoding MAAI, Fig. 1a; Supplementary Table 1). We selected sgRNAs that targeted essential protein domains and had few predicted off-target effects on the mouse genome to increase the likelihood of generating inactivating mutations (Titus *et al*. 2000; Polekhina *et al*. 2001; Lin *et al*. 2013). We identified highly active sgRNAs for all three targets by transient transfection in mouse neuroblastoma cells (Supplementary Fig. 1). Nucleases with perfectly conserved target sites and protospacer adjacent motif (PAM) sequences between the mouse and human genomes were chosen for *Hpd* and *Hgd* but the *Gstz1* target site differs by 2 bp (Fig. 1b).

All-in-one rAAV vectors expressing both an sgRNA targeting *Hpd*, *Hgd,* or *Gstz1* and hepatocyte-specific SaCas9 (*via* the TBG promoter) were constructed and produced as hepatotropic rAAV serotype 8 (rAAV8) vectors [Fig. 1b (Li *et al*. 2011; Anguela *et al*. 2013; Ran *et al*. 2015; Colella *et al*. 2018)]. Next, neonatal (2-day-old) male C57BL/6N pups were injected into the retro-orbital sinuses with 5 × 10^10^ VGs of rAAV8 targeting *Hpd, Hgd,* or *Gstz1*, then killed 40 days later. Over that period, we did not observe any changes in survival or glycemia (blood glucose levels) between vector-treated animals and saline controls (Fig. 1, c and d). However, animals treated with the *Gstz1*-targeting vector had elevated urine SA levels, and animals injected with the *Hgd*-targeting vector had detectable HGA levels as expected from their respective metabolic blocks (Fig. 1, e and f and Supplementary Tables 2 and 3).

Genomic whole-liver and kidney DNA was extracted, and Surveyor assays were used to determine indel frequencies (Guschin *et al*. 2010). Gene disruption was robust in the liver, and generally efficient, ranging from 11–47% depending on the target (Fig. 1g). Since genomic DNA was extracted from whole livers and gene editing was limited to the hepatocytes (which comprise ∼70% of the liver's mass (Palaschak *et al*. 2019)), the disruption efficiency is likely an underestimate. No editing was detected in the kidneys as expected when using rAAV8 and a liver-specific promoter to drive SaCas9 expression (Fig. 1g). Thus, this approach can partially recapitulate some biochemical phenotypes associated with mutations in the tyrosine degradation pathway, in wild-type animals, even when the metabolic block is limited to a single organ and not all hepatocytes are edited.

### In vivo inactivation of various steps in the tyrosine catabolic pathway differentially impacts metabolic outcomes and survival in HT-i mice

The impacts of liver-specific in vivo genome editing of *Hpd, Hgd,* and *Gstz1* (the gene encoding MAAI, Fig. 1a) in HT-I mice were then determined upon NTBC withdrawal. Neonatal male *Fah^−/−^* pups treated with NTBC were injected at 2 days old with rAAV8-SaCas9 targeting *Hpd, Hgd,* or *Gstz1* (or saline as a control), and NTBC was withdrawn between 4–7 weeks old (Fig. 2a). While all mice treated with the nuclease targeting *Hpd* survived and had normal weights and glycemia post-NTBC removal, saline-treated animals had to be sacrificed after ∼3 weeks, when meeting the weight loss criterion (Fig. 2, b-d). In *Hpd*-treated mice, glycemia and weight gains were normalized for over 14 weeks (Supplementary Fig. 2). In sharp contrast, *Fah^−/−^* mice treated with rAAV8-SaCas9 targeting either *Hgd* or *Gstz1* experienced sudden weight loss and hypoglycemia and died approximately 4 and 5 days post-NTBC removal, respectively (Fig. 2, b-d). Median survival significantly differed (*P* < 0.001) in these two groups when compared to the saline-treated animals.

We measured urine SA levels 24 hours prior to NTBC removal and 34 days after NTBC removal (*Hpd*) or the predicted time of death (*Hgd* and *Gstz1*). Under NTBC treatment, the urine SA levels of the different groups were broadly similar (Fig. 2e and Supplementary Table 4). The SA levels of mice treated with the *Hpd*-targeting vector significantly decreased (*P* < 0.0001 when compared to saline-injected animals) post-NTBC removal to concentrations only slightly above the detection limit (Fig. 2e and Supplementary Table 4). A group of those *Hpd*-treated mice were kept alive without NTBC for 1 year (the duration of the study) and they maintained minimal levels of SA over that period indicating that the biochemical phenotype was normalized (Supplementary Fig. 2 and Supplementary Table 5). Animals treated with the vector targeting *Hgd* showed slightly less urine SA than saline-treated animals following NTBC withdrawal (*P* < 0.05) denoting a partial blockage of the pathway (Fig. 2e and Supplementary Table 4). Notably, mice treated with the *Gstz1*-targeting vector showed increases in urine SA after drug withdrawal that were significantly higher than saline-treated controls (*P* < 0.01) (Fig. 2e and Supplementary Table 4). HGA levels in urine were undetectable in all animals except in mice treated with the vector targeting *Hgd*, which experienced a considerable increase following NTBC withdrawal (Fig. 2f and Supplementary Table 6). These biochemical changes correspond to the step blocked in the pathway (Fig. 1a).

Finally, we quantified gene disruption efficacy in hepatocytes pre- and post-NTBC withdrawal using the TIDE assay (Brinkman *et al*. 2014) (Fig. 2g). The indel profiles obtained with the TIDE assay revealed the presence of out-of-frame mutations likely to result in gene inactivation (Supplementary Fig. 1). Editing levels increased In *Hpd*-treated mice which is indicative of liver repopulation by edited hepatocytes [Fig. 2g (Pankowicz *et al*. 2016; Grompe 2017; Agudelo *et al*. 2020)]. At the time of death, livers of *Hgd*- and *Gstz1*-treated mice showed editing levels comparable to those observed in animals maintained on NTBC (Fig. 2g). Of note, the editing rates in *Fah^−/−^* animals were broadly similar to the ones observed in C57BL/6N mice (Compare Fig. 1g and Fig. 2g). Collectively, these data indicate that somatic genome editing can rewire the liver metabolism in HT-I mice to either suppress or enhance the disease.

### In vivo inactivation of various steps in the tyrosine catabolic pathway differentially impacts liver and kidney function in HT-I mice

**Fig. 5.**
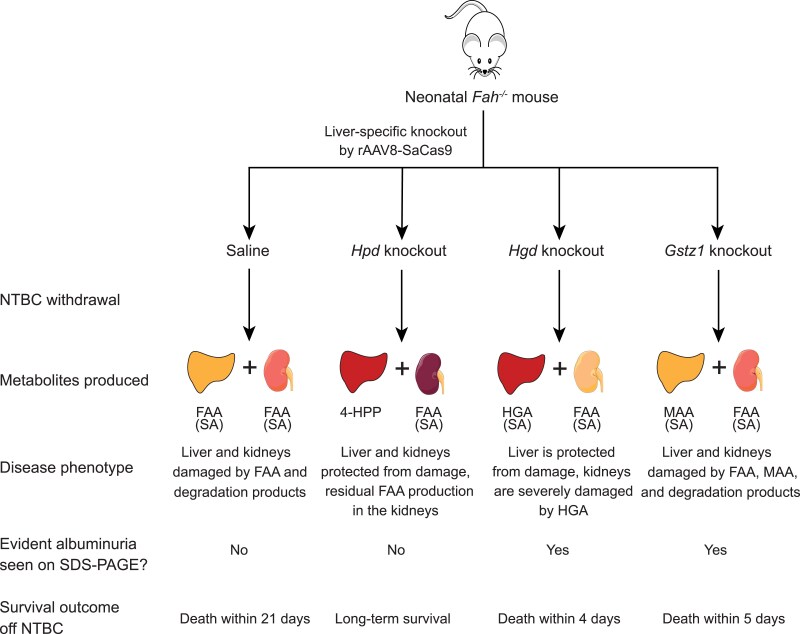
Model of metabolic rewiring in Fah−/− mice following liver-specific knockout of tyrosine catabolic enzymes. Observed disease phenotypes and survival outcomes after injecting neonatal Fah−/− pups with saline or liver-specific rAAV8-SaCas9 vectors targeting Hpd, Hgd, or Gstz1 and NTBC withdrawal. The main metabolites predicted to be produced by the liver and unedited kidneys in each condition are shown. Fumarylacetoacetate (FAA) and maleylacetoacetate (MAA) accumulation leads to non-enzymatic production of succinylacetone (SA) (see Fig. 1a).

Liver histology revealed that mice injected with the *Hgd*-targeting vector displayed substantial hepatocyte death in zones 1 and 2 with signs of apoptosis, moderate bile ductular proliferation, and portal inflammation. Hepatic steatosis and fibrosis were not detected (Fig. 5a; top). In mice injected with the *Gstz1-*targeting vector, mild lobular inflammation and significant and diffuse ballooning degeneration indicative of apoptotic hepatocyte death were observed (Fig. 5a; top). In animals injected with the *Hpd-*targeting vector, liver sections displayed moderate portal inflammation, mild ductular proliferation, and mild ballooning degeneration in zones 2 and 3, suggesting that gene-edited hepatocytes had not completely repopulated the diseased liver at the time of necropsy (Fig. 5a; top). Finally, liver histology revealed mild portal and lobular inflammation, mild ductular proliferation, glycogenated nuclei, and mixed steatosis in zone 2 in saline-injected controls (Fig. 5a; top).

As kidneys of HT-I mice are sensitive to cytotoxicity (Grompe *et al*. 1993; Grompe *et al*. 1995; Fernández-Cañón *et al*. 2002; Jacobs *et al*. 2006), we monitored kidney function and integrity. At harvest, the kidneys of mice treated with the *Hgd*-targeting vector were pale, enlarged, and displayed severe diffuse tubular damage with necrosis, which is consistent with HGA-induced toxic tubular cell injury leading to acute renal insufficiency (Fig. 5a; bottom and Fig. 5b). Those treated with the vector targeting *Gstz1* had slight tubular damage mainly in the cortical area, similarly to saline-treated controls (Fig. 5a; bottom). A group of *Hpd*-targeted mice killed 35 days after NTBC withdrawal displayed normal tubules and glomeruli, suggesting preserved kidney function (Fig. 5a; bottom). Accordingly, SDS–PAGE analysis of urine samples showed a massive increase in serum albumin (also known as albuminuria) in the animals treated with the vectors targeting *Hgd* and *Gstz1* following NTBC removal, indicative of kidney disease (Butt *et al*. 2020) (Fig. 5c). Collectively, these data indicate that liver-based editing can cause systemic effects impacting kidney function.

### Metabolic rewiring yields similar outcomes in both female and male *fah^−/−^* mice

Up to this point in the study, only male mice were used even though sexual dimorphism in response to similar rAAV-based genetic treatments had previously been reported (Davidoff *et al*. 2003; Rinn and Snyder 2005; Paulk *et al*. 2012a). Reassuringly, others have shown that inactivation of *Hpd* using CRISPR-Cas9 corrects the lethal HT-I phenotype in both female and male *Fah^−/−^* animals when delivered as plasmids via hydrodynamic tail vein injections or rAAV (Pankowicz *et al*. 2016; Ibraheim *et al*. 2018). Still, the impact of liver-specific inactivation of *Hgd* and *Gstz1* via in vivo genome editing in female *Fah^−/−^* animals has yet to be reported. To better account for sex as a biological variable and improve the quality of our study, we targeted *Hgd* and *Gstz1* in neonatal female and male *Fah^−/−^* pups side-by-side and monitored treatment outcomes. Upon NTBC withdrawal, mice of both sexes experienced sudden weight loss and hypoglycemia and met the weight loss criterion within 4 days (Fig. 3, a-c). We also observed enlarged and pale kidneys in female mice treated with the *Hgd*-targeting vector (Fig. 3d) like those observed in male *Fah^−/−^* mice treated with the same vector (Fig. 5b). In addition, gene disruption efficacy was equivalent in both groups indicating that in vivo CRISPR-Cas9 based editing using rAAV8 is similarly efficacious in our model system (Fig. 3e). Thus, liver-specific inactivation of either *Hgd* or *Gstz1* by rAAV8-SaCas9 yields similar phenotypic outcomes in both female and male *Fah^−/−^* mice.

## Discussion

Coupling rAAV-mediated in vivo gene delivery with CRISPR-Cas9 systems is a robust and rapid method to study gene functions in the somatic tissues of mice (Lau and Suh 2017; Bak *et al*. 2018; Colella *et al*. 2018; Nami *et al*. 2018; Alves-Bezerra *et al*. 2019). Here, we demonstrate that in vivo genome editing can modulate IEM-associated pathways to yield distinct phenotypes from those observed in whole-body knockout models. A limitation of this methodology is that gene disruption in somatic tissues is heterogeneous and incomplete, leaving a fraction of cells with altered functions that can affect the phenotype.

In *Fah^−/−^* mice of both sexes, metabolic rewiring via in vivo editing recapitulated several hallmarks of HT-I. First, targeting *Hpd* rescued the lethal phenotype, as previously observed (Endo *et al*. 1997; Pankowicz *et al*. 2016; Ibraheim *et al*. 2018; Agudelo *et al*. 2020). This rescue was maintained for at least 1 year post-NTBC removal. An increase in the editing level occurred over time in these mice likely due to the potent advantage of corrected hepatocytes following NTBC removal (Pankowicz *et al*. 2016; Grompe 2017; Agudelo *et al*. 2020). We also observed that liver-specific targeting of *Gstz1* in *Fah^−/−^* mice resulted in pronounced SA excretion and rapid death as in conventional double mutant *Fah*^−/−^  *Gstz1*^−/−^ mice (Fernández-Cañón *et al*. 2002) (Fig. 4). Interestingly, targeting *Hgd* resulted in the opposite phenotype compared to that observed in a classical knockout mouse model and when an FAH inhibitor was used to select *Hgd^−/−^* hepatocytes transplanted into wild-type recipient mice (Manning *et al*. 1999; Paulk *et al*. 2012b). While whole-body *Fah^−/−^ Hgd^−/−^* mice were protected from liver and renal damage, liver-specific inactivation of *Hgd* via in vivo editing in *Fah^−/−^* mice resulted in rapid death likely caused by kidney failure. This apparent discrepancy could be attributed to the fact that liver-specific *Hgd* disruption followed by NTBC withdrawal resulted in the rapid production of a massive amount of HGA, which is known to cause renal damage to *Fah^−/−^* mice (Sun *et al*. 2000; Jacobs *et al*. 2006). This HGA can be processed in the kidneys by active HGD and MAAI leading to the local accumulation of FAA and SA since FAH is not present in this organ in the *Fah^−/−^* mice (Fig. 4). Previous work had shown that phenotypic rescue of HT-I in mice can occur by *Hgd* inactivation, an in vivo suppressor mutation (Manning *et al*. 1999). In these spontaneous revertants, liver-function tests were normal but kidneys were pale and enlarged and showed extensive tubular damage (Manning *et al*. 1999). In this original work, these data were not shown but they are reminiscent of our observations. It appears that the main difference is that our system rapidly produced a higher fraction of hepatocytes inactivated for *Hgd*, which created a bolus of HGA following sudden NTBC removal, which prevented any adaptation and caused acute renal failure (Fig. 4). In HT-I, there is evidence that negative feedback loops can inhibit or down-regulate upstream enzymes in the tyrosine degradation pathway. For example, in *Fah^−/−^* mice, TAT mRNA levels are markedly reduced without NTBC treatment (Grompe *et al*. 1995) (Fig. 1a). In humans, HPD's enzymatic activity is greatly reduced in patients with HT-I compared to healthy controls (La Du 1967; Lindblad *et al*. 1977). These compensatory mechanisms may partially protect liver and kidney cells from the toxic accumulation of FAA and SA and may have been bypassed by the sudden perturbations created by the editing process and NTBC withdrawal.

We propose a model of the metabolic interplay between the edited liver and the non-edited kidneys in *Fah^−/−^* mice following NTBC withdrawal (Fig. 4). Of note, this model does not exclude the possibility that circulating HGA can also reenter non-targeted, HGD-expressing hepatocytes, and cause their rapid death (Vogel *et al*. 2004; Hughes *et al*. 2019). Irrespective of the physiological mechanism, the differences between our observations and those of whole-body knockout models highlight the importance of using tissue-specific genome editing in animal models of human genetic disorders to investigate its systemic impacts. It has recently been shown that metabolic pathway rewiring following liver-directed CRISPR-Cas9 knockout can be used to rescue glutaric acidemia type 1, an inborn error of metabolism affecting the lysine degradation pathway (Barzi *et al*. 2023). In vivo genome editing is thus a promising approach that could be used to dissect metabolic pathways affected in other IEMs.

Other than the limitation imposed by the impossibility of editing all hepatocytes within the liver, even in mice, there may be major differences in phenotypes between humans and mice deficient in the same gene product. A prime example is that HT-I mice have a neonatal lethal phenotype and are not tyrosinemic (i.e. they do not have elevations of plasma tyrosine) (Grompe *et al*. 1993), unless treated with NTBC (Grompe *et al*. 1995), contrary to humans with tyrosinemia type I. Our studies have also been performed in neonates which may have differences in liver functions compared to adult mice.

## Supplementary Material

iyae139_Supplementary_Data

## Data Availability

The authors affirm that all data necessary for confirming the conclusions of this article are represented fully within the article and its tables and figures. Supplemental material available at GENETICS online.
