# Supplementary material for: In vivo dissection of the mouse tyrosine catabolic pathway with CRISPR-Cas9 identifies modifier genes affecting hereditary tyrosinemia type 1
Source: Genetics. 2024 Aug 23;228(2):iyae139. doi: 10.1093/genetics/iyae139 (PMC11457941; doi:10.1093/genetics/iyae139)
Supplement: iyae139_Supplementary_Data [file iyae139_supplementary_data.pdf]

SUPPLEMENTARY FIGURES AND TABLES

In vivo dissection of the mouse tyrosine catabolic pathway with CRISPR-Cas9 identifies modifier genes affecting hereditary tyrosinemia type 1

Jean-François Rivest[1,2], Sophie Carter[1,2], Claudia Goupil[1,2], Pénélope Antérieux[1,2] Denis Cyr[3], Roth-Visal Ung[1], Dorothée Dal Soglio[4], Fabrice Mac-Way[1], Paula J. Waters[3], Massimiliano Paganelli[4], and Yannick Doyon[1,2].

[1]Centre Hospitalier Universitaire de Québec Research Center and Faculty of Medicine, Laval University, Québec City, QC, G1V 4G2, Canada.

[2]Université Laval Cancer Research Centre, Québec City, QC, G1V 0A6, Canada.

[3] Medical Genetics Service, Dept. Laboratory Medicine and Dept. Pediatrics, Centre Hospitalier Universitaire de Sherbrooke (CHUS), Sherbrooke, QC, J1H 5N4, Canada.

[4]Sainte-Justine University Hospital Center, Université de Montréal, Montréal, QC, H3T 1C5, Canada.

Address correspondence to:
Yannick Doyon, Ph.D.
Centre de recherche du CHU de Québec – Université Laval
2705, boulevard Laurier, T-3-67
Québec, QC G1V 4G2
CANADA
Tel: 418-525-4444 ext. 46264
Email: Yannick.Doyon@crchudequebec.ulaval.ca

SUPPLEMENTARY FIGURES

Supplementary Fig. 1 | Screening for active SaCas9 sgRNAs in mouse Neuro-2a cells and representative *in vivo* indels profiles

Supplementary Fig. 2 | Long-term follow-up of *Hpd*-targeted mice after removing NTBC

Supplementary Fig. 3 | Purified rAAV8 vectors used in this study

SUPPLEMENTARY TABLES

Supplementary Table 1 | SaCas9 guide RNA (spacer) sequences

Supplementary Table 2 | Raw urine succinylacetone quantification data from C57BL/6N mice

Supplementary Table 3 | Raw data from urine homogentisic acid quantification from C57BL/6N mice

Supplementary Table 4 | Raw urine succinylacetone quantification data from *Fah*$^{-/-}$ mice

Supplementary Table 5 | Raw data from urine succinylacetone quantification in *Hpd*-targeted *Fah*$^{-/-}$ mice 1 year after NTBC removal

Supplementary Table 6 | Raw urine homogentisic acid quantification data from *Fah*$^{-/-}$ mice

Supplementary Table 7 | PCR primers used in Surveyor and TIDE assays and their amplicon sizes

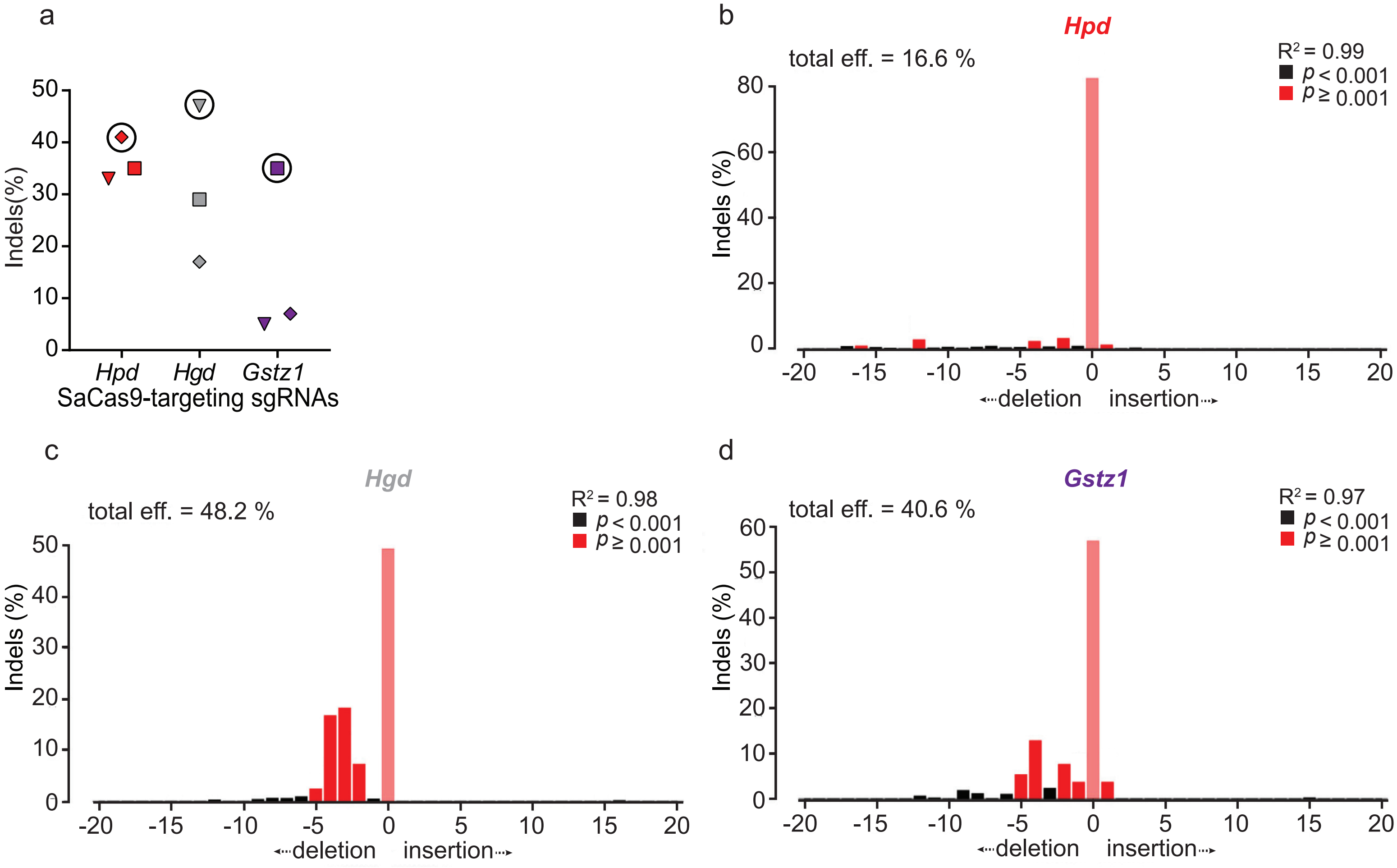

Supplementary Fig. 1. Screening for active SaCas9 sgRNAs in mouse Neuro-2a cells and representative *in vivo* indels profiles (a) Surveyor assays to determine the activities of sgRNAs targeting SaCas9 to *Hpd*, *Hgd*, and *Gstz1*. Neuro-2a cells were transiently transfected with 500ng of a single vector expressing SaCas9 and each sgRNA. Each symbol represents a different sgRNA. Surveyor assays were performed 3 days later to determine the frequency of SaCas9-induced insertions and deletions (indels). A vector encoding EGFP was used as a negative control (not shown). Circles indicate the sgRNAs chosen for *in vivo* studies. (b-d) Representative TIDE analyses of *Fah*$^{-/-}$ mice injected with rAAV8-SaCas9 vectors targeting *Hpd* (b), *Hgd* (c), and *Gstz1* (d) and sacrificed at 30 days of age with continuous NTBC treatment as described in Fig. 2g.

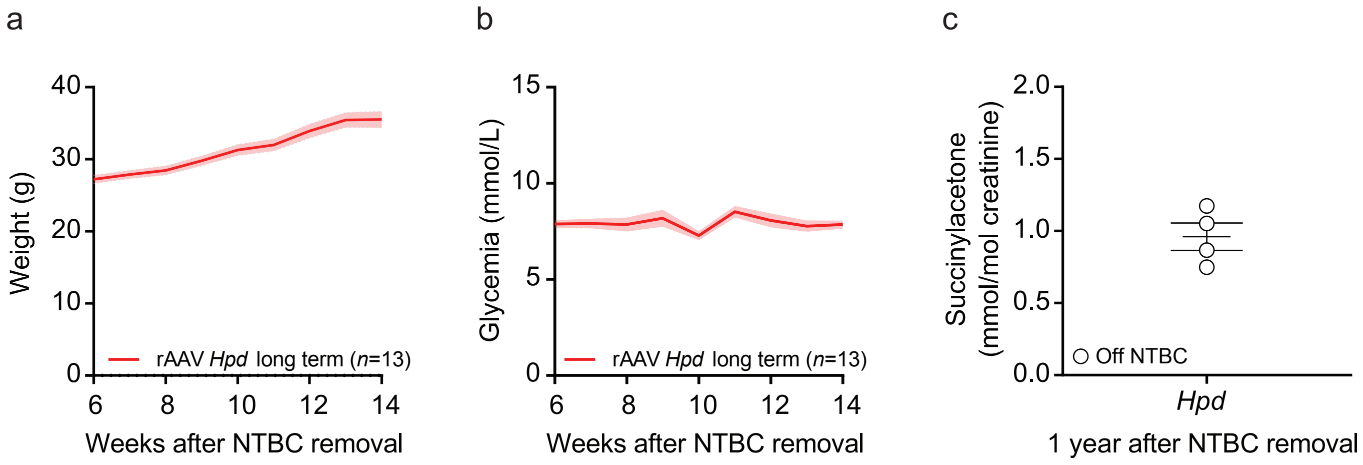

Supplementary Fig. 2. Long-term follow-up of *Hpd*-targeted *Fah*$^{-/-}$ mice after removing NTBC (a) Long-term weights and (b) glycemia of the mice taken off NTBC in Fig.2, from weeks 6–14 post-removal. (c) One year after NTBC removal, urine samples were collected over 24 hours using metabolic cages and succinylacetone levels were measured. Also indicated are the mean and the standard error of the mean (SEM). The detection limit was 0.1 mmol/mol creatinine. Raw data are presented in Supplementary Table 6.

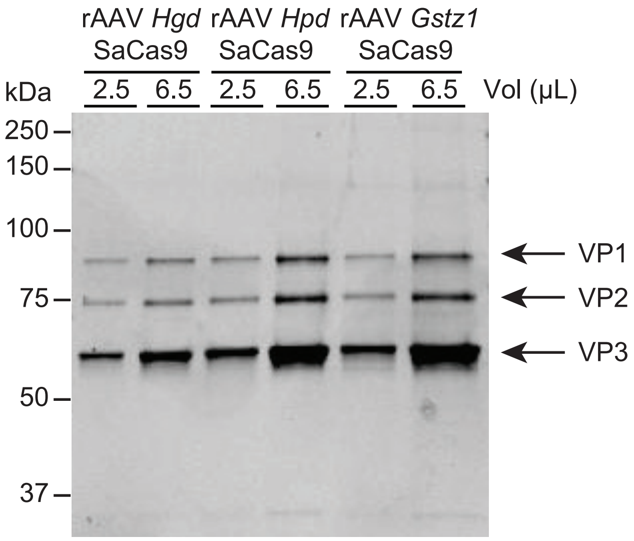

Supplementary Fig. 3. Purified rAAV8 vectors used in this study. Fixed volumes of the purified rAAV8-SaCas9 vectors used in the study were resolved on mini-PROTEAN TGX Stain-Free Gels. The molecular weights (kDa) of protein standards are indicated. The three rAAV capsid proteins VP1, VP2 and VP3 are indicated by an arrow.

Supplementary Table 1 | SaCas9 guide RNA (spacer) sequences

Gene	Exon	Target size (bp)	PAM	Target	Indels in the Surveyor assay (%)
Hpd	3	20	CCGAGT	GATTGCCAACCCAGAAGGTCA	35
	7	20	GTGAGT	**GAGTTTGCTGTGCTGCAGACG**	**41**
	10	20	CGGAGT	GGACGACACGCAGGTGCACA	33
Hgd	9	20	TTGAGT	**GGAGGTCTATGGTGTCCACT**	**47**
	12	21	GGGAGT	GTGTCATCTTCCCACCTCGGTG	29
	13	21	GTGGAT	GTCACTATGAGGCAAAGCAAG	17
Gstz1	4	20	GTGAGT	**GAATCACCATTGTCCAGTCA**	**35**
	5	20	GTGGGT	GCGCACGATGGCTCTTTTCT	5
	8	20	ATGGAT	GTACTAAGCACACATCAGCC	7

Target sequences for *Hpd*, *Hgd* and *Gstz1* were selected using CRISPOR. We selected sgRNAs with high predicted activity and low predicted off-target scores. When required, the sgRNA sequence at position 1 was changed to 'G' to meet the transcription initiation requirements of the human U6 promoter (indicated in bold red). The target sequences in bold were chosen for *in vivo* studies. Underlined blue nucleotides indicate mismatches with the human genome sequence for one of the chosen targets.

Supplementary Table 2 | Raw urine succinylacetone quantification data from C57BL/6N mice

Group	Units	Cage 1	Cage 2
Hpd	mmol/mol creatinine (number of animals per cage)	0.23 (2)	
Hgd		BDL (3)	BDL (3)
Gstz1		76.67 (2)	24.07 (2)

Urine was collected overnight before and at different time points after NTBC removal from groups of 2–3 mice as described in Fig. 1. Number of mice per cage is indicated in parenthesis. Succinylacetone levels were quantified by GC-MS. BDL: Below detection limit

Supplementary Table 3 | Raw data from urine homogentisic acid quantification from C57BL/6N mice

Groups	Units	Cage 1	Cage 2	Cage 3	Cage 4
Hpd	mmol/mol creatinine (number of animals per cage)	BDL (2)			
Hgd		5 (3)	35 (3)	6 (2)	4 (2)
Gstz1		BDL (2)			

Urine was collected overnight before and at different time points after NTBC removal from groups of 2–3 mice as described in Fig. 1. Number of mice per cage is indicated in parenthesis. Homogentisic acid levels were quantified by GC-MS. BDL: Below detection limit

Supplementary Table 4 | Raw urine succinylacetone quantification data from *Fah*$^{-/-}$ mice

Group	Units	On NTBC				Off NTBC			
		Cage 1	Cage 2	Cage 3	Cage 4	Cage 1	Cage 2	Cage 3	Cage 4
Saline	mmol/mol creatinine (number of animals per cage)	8.05 (4)	20.18 (3)	9.59 (3)	9.24 (5)	74.77 (3)	53.16 (2)		
Hpd		5.49 (4)	7.58 (5)	7.09 (3)		0.51 (3)	0.51 (4)	0.50 (3)	0.52 (4)
Hgd		5.40 (5)	5.47 (4)	12.17 (2)		24.15 (3)	16.77 (5)		
Gstz1		10.06 (3)	11.97 (3)	11.18 (5)		82.23 (3)	133.35 (3)	117.28 (2)	

Urine was collected overnight before and at different time points after NTBC removal from groups of 2–5 mice as described in Fig. 2. Number of mice per cage is indicated in parenthesis. Succinylacetone levels were quantified by GC-MS.

Supplementary Table 5 | Raw data from urine succinylacetone quantification in *Hpd*-targeted *Fah*$^{-/-}$ mice 1 year after NTBC removal

Group	Units	Cage 1	Cage 2	Cage 3	Cage 4
Hpd long-term	mmol/mol creatinine (number of animals per cage)	1.17 (3)	1.05 (3)	0.75 (4)	0.87 (3)

Urine was collected overnight before and at different time points after NTBC removal from groups of 3–4 mice as described in Supplementary Fig. 2. Number of mice per cage is indicated in parenthesis. Succinylacetone levels were quantified by GC-MS.

Supplementary Table 6 | Raw urine homogentisic acid quantification data from *Fah*$^{-/-}$ mice

Group	Units	On NTBC			Off NTBC		
		Cage 1	Cage 2	Cage 3	Cage 1	Cage 2	Cage 3
Saline	mmol/mol creatinine (number of animals per cage)	BDL (4)	BDL (3)		BDL (3)	BDL (2)	
Hpd		BDL (4)	BDL (5)	BDL (3)	BDL (3)	BDL (4)	BDL (3)
Hgd		22 (5)	4 (4)		1613 (3)	1440 (5)	
Gstz1		BDL (3)	BDL (3)	BDL (5)	BDL (3)	BDL (3)	BDL (2)

Urine was collected overnight before and at different time points after NTBC removal from groups of 2–5 mice as described in Fig. 2. Number of mice per cage is indicated in parenthesis. Homogentisic acid levels were quantified by GC-MS. BDL: Below detection limit

Supplementary Table 7 | PCR primers used in Surveyor and TIDE assays and their amplicon sizes

Target	Primer	Size (bp)
Hpd exon 3 Forward	GTCACCCATACTGTTCTCACGTA	466
Hpd exon 3 Reverse	CAAGGTTCCAAAGTGCCAGTCC	
Hpd exon 7 Forward	GCAGGCGCAGTGCCCAAGACAC	498
Hpd exon 7 Reverse	CAGCACATGCCCAGGTCACATGG	
Hpd exon 10 Forward	GTGTAACGGGTGTATGCTCAATG	452
Hpd exon 10 Reverse	GTGATGATGTCTTCCGTCTTGAG	
Hgd exon 9 Forward	TGAGTTGTGGCTAACTGGGG	420
Hgd exon 9 Reverse	AGGCAGGCATTTTGTCTAAGGA	
Hgd exon 12 Forward	CTGTCACTTGAAAGCACCCT	451
Hgd exon 12 Reverse	TCACACTCTCCCAGCCTGTC	
Hgd exon 13 Forward	GTGCTATTGTGGAATAGTTG	412
Hgd exon 13 Reverse	TCCTCACTCCACCTCTGTGA	
Gstz1 exon 4 Forward	GACCACAGTAAAGAGTACAGGGA	400
Gstz1 exon 4 Reverse	GCTTGGTCACTTGTAGGTTAGTT	
Gstz1 exon 5 Forward	GGAGTTTGCTGCCTCTCCCCTC	505
Gstz1 exon 5 Reverse	GCTACAGAGCAGATGACCAGGAG	
Gstz1 exon 8 Forward	GGGAAACTTGACATGGGAGAAAT	393
Gstz1 exon 8 Reverse	CAGTTGATAATGGCCTGGTGTAG	
